# *SLC39A13* Defines Myofibroblastic Activation and Immunosuppressive Tumor Microenvironment in Head and Neck Squamous Cell Carcinoma

**DOI:** 10.3390/curroncol33050292

**Published:** 2026-05-18

**Authors:** Hideyuki Takahashi, Hiroyuki Hagiwara, Hiroe Tada, Miho Uchida, Toshiyuki Matsuyama, Kazuaki Chikamatsu

**Affiliations:** Department of Otolaryngology-Head and Neck Surgery, Gunma University Graduate School of Medicine, 3-39-22 Showa-machi, Maebashi 371-8511, Gunma, Japantikamatu@gunma-u.ac.jp (K.C.)

**Keywords:** head and neck squamous cell carcinoma, cancer-associated fibroblasts, zinc transporters, *SLC39A13*, tumor microenvironment, immune suppression, single-cell RNA sequencing

## Abstract

Zinc transport plays a critical role in cellular signaling, but its function in the tumor microenvironment remains poorly understood. We aimed to investigate the role of zinc transporters in cancer-associated fibroblasts in head and neck squamous cell carcinoma. Single-cell RNA sequencing data were analyzed to evaluate zinc transporter expression across tumor cell populations, and bulk RNA sequencing of primary fibroblast cultures was used for validation. Clinical relevance was assessed using transcriptomic and survival data from a large patient cohort. We found that zinc transporter expression, particularly *SLC39A13*, was enriched in fibroblasts and strongly associated with myofibroblastic activation signatures, including extracellular matrix remodeling and TGFβ signaling. Fibroblasts with high *SLC39A13* expression were linked to immunosuppressive tumor environments characterized by reduced cytotoxic T-cell infiltration and increased immunosuppressive cells. Clinically, *SLC39A13* expression was associated with poor progression-free survival and remained an independent prognostic factor. These findings suggest that zinc transporter-mediated pathways play a key role in stromal activation and immune regulation, highlighting *SLC39A13* as a potential therapeutic target in head and neck squamous cell carcinoma.

## Simple Summary

Head and neck squamous cell carcinoma is influenced not only by cancer cells but also by surrounding support cells called fibroblasts. These cells can promote tumor growth and suppress immune responses, but the mechanisms controlling their behavior remain unclear. In this study, we investigated the role of zinc transport within fibroblasts. We found that a specific zinc transporter, *SLC39A13*, is highly active in these cells and is linked to a state that promotes tissue remodeling and weakens anti-tumor immunity. Tumors with high *SLC39A13* activity showed reduced infiltration of immune cells that normally attack cancer and were associated with poorer clinical outcomes. These findings suggest that zinc transport plays an important role in shaping the tumor environment. Targeting this pathway may help improve treatment strategies by modifying both tumor-supporting cells and immune responses.

## 1. Introduction

Head and neck squamous cell carcinoma (HNSCC) is a biologically heterogeneous disease characterized by diverse clinical outcomes and variable responses to therapy [1,2,3]. Despite advances in multimodal treatment, including surgery, radiotherapy, and systemic therapies, the prognosis of patients with advanced HNSCC remains unsatisfactory [3,4,5,6]. In addition to tumor-intrinsic factors, increasing evidence highlights the critical role of the tumor microenvironment (TME) in shaping tumor progression and therapeutic resistance [7,8,9].

Cancer-associated fibroblasts (CAFs) are a major stromal component of the TME and are known to contribute to tumor progression, extracellular matrix remodeling, and immune regulation [10,11,12]. CAFs exhibit substantial functional heterogeneity and can be broadly classified into subtypes such as myofibroblastic CAFs (myCAFs) and inflammatory CAFs (iCAFs) [11,13,14,15]. In particular, myCAFs are characterized by strong extracellular matrix production and TGFβ-driven activation and have been implicated in tumor progression and immune suppression [11,13,16]. However, the molecular mechanisms underlying CAF heterogeneity and functional specialization remain incompletely understood.

Zinc is an essential trace element involved in numerous biological processes, including cell proliferation, differentiation, and immune regulation [17,18,19]. Intracellular zinc homeostasis is tightly controlled by two major transporter families: ZIP (SLC39A) transporters, which increase cytosolic zinc levels, and ZnT (SLC30A) transporters, which reduce cytosolic zinc [17,18]. Dysregulation of zinc transport has been implicated in various cancers, where it influences tumor growth, metastasis, and immune responses [20,21,22,23]. However, the role of zinc transporters in the tumor microenvironment, particularly in CAF biology, remains largely unexplored.

In this study, we performed a comprehensive analysis of zinc transporter expression in HNSCC using single-cell RNA sequencing, bulk RNA sequencing of primary CAFs, and publicly available transcriptomic datasets. We aimed to elucidate the cell type-specific expression patterns of ZIP transporters, their association with CAF functional states, and their clinical and immunological relevance. Our findings identify specific ZIP transporters, particularly *SLC39A13*, as key regulators of myofibroblastic activation programs in CAFs and demonstrate their association with immunosuppressive tumor microenvironments and poor clinical outcomes in HNSCC. Our study highlights a fibroblast-specific role of *SLC39A13* linking intracellular zinc regulation to stromal activation and immune modulation.

## 2. Materials and Methods

### 2.1. Acquisition of the GSE164690 Dataset

Single-cell RNA sequencing (scRNA-Seq) data were obtained from the Gene Expression Omnibus (GEO) database (accession number GSE164690). The dataset consists of freshly resected primary head and neck squamous cell carcinoma specimens, including both human papillomavirus (HPV)-positive and HPV-negative cases. It comprises CD45-positive immune cells and CD45-negative non-immune cells collected from 14 tumor samples. Clinical information for these samples is provided in Supplementary Table S1. All preprocessing and downstream analyses were performed using the Seurat R package (version 5).

### 2.2. Processing of scRNA-Seq Data

Cells expressing fewer than 100 genes were filtered out prior to analysis. The remaining data were normalized using a global scaling approach with a scale factor of 10,000, followed by identification of the top 2000 highly variable genes for downstream analysis. Dimensionality reduction was performed using Uniform Manifold Approximation and Projection (UMAP), and cells were clustered with the FindClusters function at a resolution of 0.2, yielding 16 clusters. Cluster-specific marker genes were identified using the FindAllMarkers function.

To evaluate cell-type enrichment, mean normalized expression values for each cluster were calculated and analyzed using the xCell R package (version 1.1.0). Zinc transporter activity was quantified by calculating ZIP and ZnT scores, defined as the average normalized expression of genes belonging to the SLC39A and SLC30A families, respectively. No weighting was applied to individual genes to maintain interpretability and avoid assumptions regarding their relative contributions. These scores were computed at the single-cell level and subsequently summarized across cell populations for downstream analyses.

Fibroblast-specific patient-level pseudo-bulk profiles were generated by averaging log-normalized expression values across fibroblasts from each patient. Based on these patient-level mean expression matrices, functional signature scores were calculated as the mean expression of predefined gene sets. The myCAF-ECM signature was defined as the average expression of *COL1A1*, *COL1A2*, *ACTA2*, *TAGLN*, and *CTGF*. The iCAF signature was defined as the average expression of *IL6*, *CXCL12*, *CXCL8*, and *LIF*. The TGFβ response signature was defined as the average expression of *SERPINE1*, *CTGF*, and *SMAD7*. The MMP score was calculated as the mean expression of *MMP2*, *MMP3*, *MMP9*, and *MMP14*, and the TIMP score as the mean expression of *TIMP1* and *TIMP2*. The MMP–TIMP ratio was then calculated as log ((MMP score + ε)/(TIMP score + ε)) to avoid division by zero (ε = 10^−6^). Correlations between ZIP transporter expression and each signature were evaluated at the patient level using Spearman’s rank correlation coefficient.

Subsequently, 5294 fibroblasts were extracted from the integrated Seurat object and re-clustered independently. CAF subtype signatures were derived from the top 20 differentially expressed genes for myCAF, iCAF1, iCAF2, and antigen-presenting CAF (apCAF) obtained from our previous study (Supplementary Table S2) [24]. Per-cell subtype scores were calculated using Seurat’s AddModuleScore. Re-clustering-derived fibroblast clusters were then evaluated based on both the mean subtype scores and cluster marker genes identified by FindAllMarkers function. Cells outside these annotated clusters were excluded from downstream CAF subtype analyses.

### 2.3. Isolation and Culture of CAFs

Primary CAFs were established from tumor tissues obtained from 10 patients with newly diagnosed HNSCC, including five cases each of oropharyngeal and hypopharyngeal cancer, as previously described [25,26]. Clinical characteristics of the patients are summarized in Supplementary Table S3. Tumor specimens were mechanically dissociated into small fragments (approximately 1–3 mm^3^) and cultured under sterile conditions. Cells were maintained in Dulbecco’s modified Eagle medium DMEM supplemented with 10% fetal bovine serum, 2 mM L-glutamine, and antibiotics. Following serial passaging, adherent fibroblast-like cells were selectively expanded. Cell identity was confirmed by flow cytometric analysis using established CAF markers, including fibroblast activation protein (FAP), CD90, and α-smooth muscle actin (α-SMA). Only early-passage cells (passage < 10) were used for downstream experiments. All procedures were conducted in accordance with institutional guidelines and were approved by the Institutional Review Board of Gunma University (approval number HS2017-152). Written informed consent was obtained from all patients prior to sample collection.

### 2.4. Bulk RNA Sequencing and Data Processing

Total RNA was isolated from cultured CAFs using the FastGene RNA Premium Kit (Nippon Genetics, Tokyo, Japan) following the manufacturer’s instructions. RNA integrity was evaluated with an Agilent Bioanalyzer, and only high-quality samples (RNA integrity number > 9.7) were selected for library preparation. Libraries for RNA sequencing were prepared using the KAPA mRNA HyperPrep Kit (Kapa Biosystems, Wilmington, MA, USA) together with SeqCap adapters (Roche Sequencing Solutions, Pleasanton, CA, USA). Sequencing was carried out on an Illumina NextSeq500 platform to generate paired-end reads of 43 base pairs. Raw sequencing reads were mapped to the human reference genome (hg19) using STAR (version 2.5.3a). Gene-level expression quantification was performed with RSEM (version 1.3.3). Expression values were log2-transformed prior to downstream analysis. For sample stratification, unsupervised hierarchical clustering was conducted based on z-score–normalized expression of *SLC39A7*, *SLC39A8*, *SLC39A13*, and *SLC39A14*. Heatmaps were generated using the pheatmap package in R.

### 2.5. ZIP Transporter-Based Stratification Defines Distinct Stromal and Immune Phenotypes in HNSCC

Transcriptomic data (Illumina HiSeq RNA-Seq V2 normalized counts), together with clinical annotations for 520 patients with HNSCC, were obtained from FireBrowse (http://firebrowse.org/), including 97 HPV-positive and 423 HPV-negative cases. The relationship between gene expression and clinical outcomes was assessed using Cox proportional hazards models. Cutoff values for survival analyses were determined by receiver operating characteristic (ROC) curve analysis. Univariate analyses were initially conducted for overall survival (OS) and progression-free survival (PFS), and variables with *p* < 0.05 were subsequently included in multivariate Cox regression models. For OS, the multivariate model included primary lesion, T factor, N factor, M factor, TNM stage, *SLC39A13* expression, and *SLC39A14* expression. For PFS, the multivariate model included T factor, TNM stage, *SLC39A7* expression, and *SLC39A13* expression. Survival curves were generated using the Kaplan–Meier method and compared by the log-rank test. Based on the z-scores of *SLC39A7* and *SLC39A13* expression, patients were grouped into three clusters using hierarchical clustering (hclust function in R), representing distinct ZIP expression patterns.

The tumor immune microenvironment was estimated using CIBERSORTx (https://cibersortx.stanford.edu, accessed on 15 July 2026; Stanford University, Stanford, CA, USA), applying the LM22 signature matrix to infer the relative abundance of 22 immune cell populations from bulk RNA-Seq data. Immune cell fractions were compared across ZIP-defined clusters to characterize differences in immune infiltration patterns.

To further explore biological differences between clusters, gene set enrichment analysis (GSEA; version 4, Broad Institute) was performed using ranked gene expression data. Enrichment scores were calculated using the Hallmark gene sets, and results were reported as normalized enrichment scores (NESs) along with the nominal *p*-values and false discovery rate (FDR) q-values.

### 2.6. Statistical Analysis

All statistical analyses were conducted using R software (version 4.3.1). Differences in continuous variables were evaluated by one-way analysis of variance (ANOVA) followed by Tukey’s post hoc test, whereas categorical variables were compared using the chi-square test. All statistical tests were two-sided, and a *p*-value of less than 0.05 was considered statistically significant.

## 3. Results

### 3.1. Single-Cell Transcriptomic Analysis Identifies Fibroblasts as the Major ZIP-Expressing Cell Population in HNSCC

**Figure 1:**
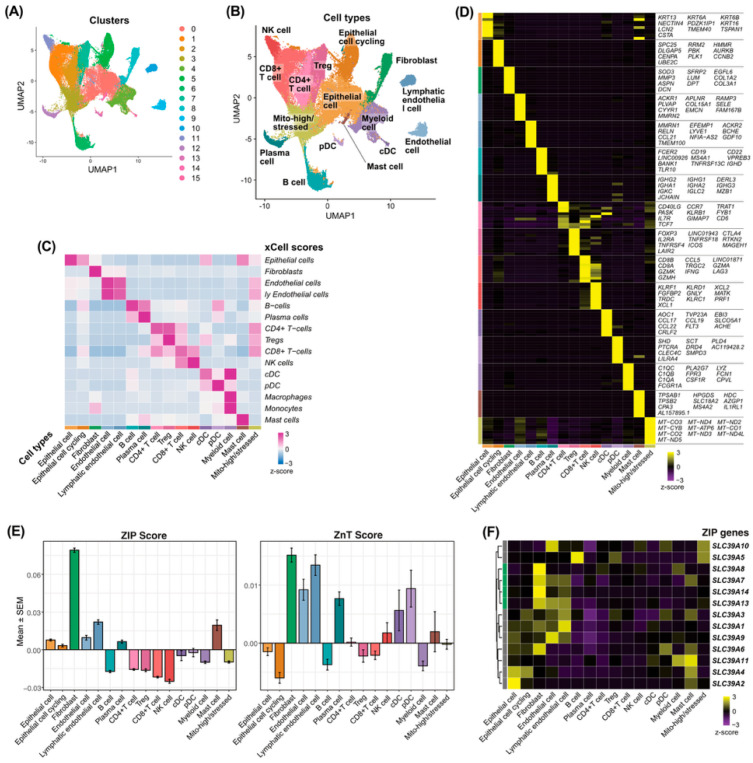
Single-cell transcriptomic profiling of HNSCC identified fibroblasts as the major ZIP-expressing cell population. (**A**) UMAP visualization of 16 cell clusters identified from the GSE164690 dataset. (**B**) UMAP showing cell type annotation based on canonical marker genes. (**C**) Heatmap showing xCell enrichment scores across annotated cell types. (**D**) Heatmap showing the expression of representative marker genes across cell types. (**E**) Bar plots showing ZIP and ZnT scores across major cell types. (**F**) Heatmap showing the expression of ZIP transporter genes across cell populations.

To characterize zinc transporter expression across the tumor microenvironment, we analyzed the GSE164690 scRNA-Seq dataset. Unsupervised clustering identified 16 distinct cell populations (Figure 1A). Cell types were annotated based on canonical marker genes and xCell enrichment scores (Figure 1B–D). Among these cell types, fibroblasts exhibited the highest ZIP score compared with other populations, whereas ZnT scores showed less cell type-specific enrichment (Figure 1E). Expression profiling of individual ZIP transporters revealed that several genes, including *SLC39A7*, *SLC39A8*, *SLC39A13*, and *SLC39A14*, were preferentially expressed in fibroblasts (Figure 1F; Supplementary Figure S1A). In contrast, no ZnT transporter showed preferential expression in fibroblasts (Supplementary Figure S1B,C).

### 3.2. ZIP Transporters Are Associated with Clinicopathological Features and Myofibroblastic Activation Programs in CAFs

**Figure 2:**
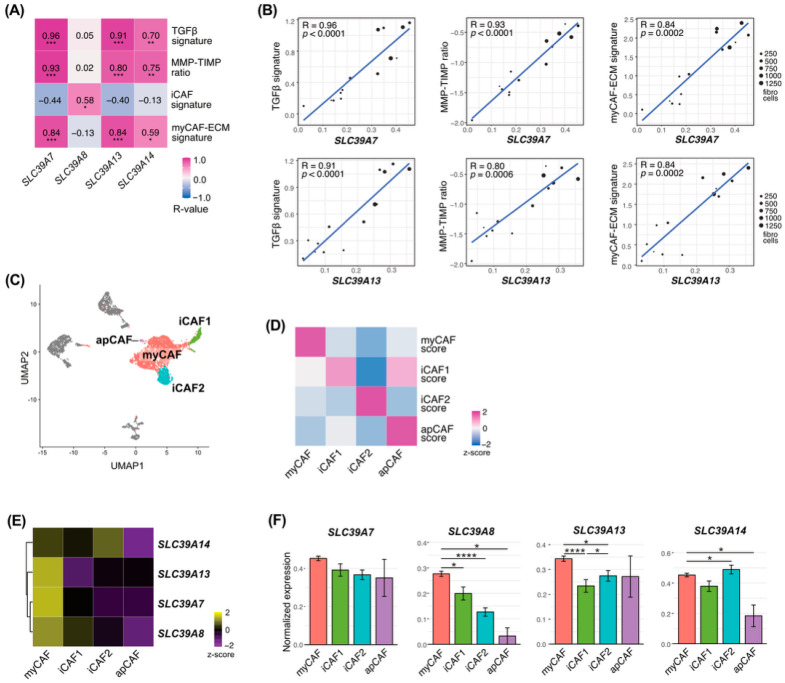
ZIP transporter expression is associated with myofibroblastic activation programs in CAFs. (**A**) Heatmap showing correlations between ZIP transporter expression and stromal activation signatures, including TGFβ signature, MMP–TIMP ratio, iCAF signature, and myCAF-ECM signature. (**B**) Scatter plots showing correlations between *SLC39A7* or *SLC39A13* expression and TGFβ signature, MMP–TIMP ratio, and myCAF-ECM signature at the patient level. (**C**) UMAP visualization of reclustered fibroblasts annotated as CAF subtypes, including myCAF, iCAF1, iCAF2, and apCAF. (**D**) Heatmap showing CAF subtype module scores across annotated CAF subtypes. (**E**) Heatmap showing ZIP transporter gene expression across CAF subtypes. (**F**) Bar plots showing normalized expression of ZIP transporter genes across CAF subtypes. *, *p* < 0.05; **, *p* < 0.01; ***, *p* < 0.001, **** *p* < 0.0001.

Given that *SLC39A7*, *SLC39A8*, *SLC39A13*, and *SLC39A14* were preferentially expressed in fibroblasts, we focused subsequent analyses on these four ZIP transporter genes. We examined the associations between ZIP transporter expression and clinicopathological features in CAFs (Table 1). Both *SLC39A13* and *SLC39A14* expression showed significant associations with multiple clinical parameters, whereas other ZIP transporters demonstrated weaker or non-significant relationships. To investigate the functional relevance of ZIP transporter expression in fibroblasts, we examined the correlations between ZIP gene expression and stromal activation signatures. Among these four transporters, *SLC39A7* and *SLC39A13* expression showed strong positive correlations with TGFβ response, MMP–TIMP ratio, and myCAF-ECM signatures (Figure 2A,B; Supplementary Figure S2A).

Re-clustering of fibroblasts identified four CAF subtypes (myCAF, iCAF1, iCAF2, and apCAF) (Figure 2C–D, Supplementary Figure S2B). Notably, *SLC39A7*, *SLC39A8*, and *SLC39A13* were predominantly expressed in myCAF populations, whereas their expression was relatively lower in other CAF subtypes (Figure 2E,F).

### 3.3. Bulk RNA Sequencing of Primary CAFs Reveals Distinct ZIP-Associated Transcriptional Programs

**Figure 3:**
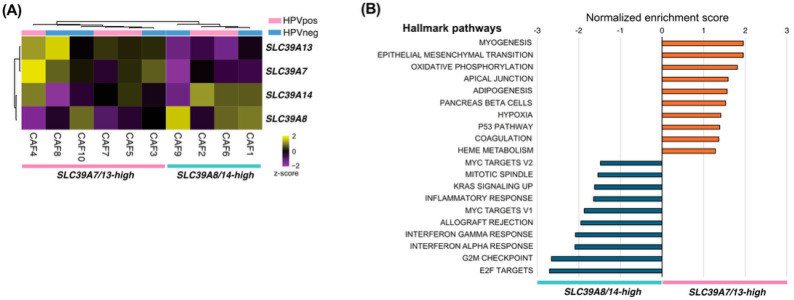
Bulk RNA sequencing of primary CAFs revealed distinct ZIP-associated transcriptional programs. (**A**) Unsupervised hierarchical clustering of CAF samples based on ZIP transporter expression. (**B**) Gene set enrichment analysis of ZIP-defined groups showing the enrichment of selected biological pathways.

To validate these findings, bulk RNA sequencing was performed on primary CAF cultures derived from HNSCC tumors. Unsupervised clustering based on ZIP transporter expression separated samples into two groups characterized by high SLC39A7/SLC39A13 expression or high SLC39A8/SLC39A14 expression (Figure 3A). Gene set enrichment analysis revealed that the SLC39A7/SLC39A13-high group was significantly enriched in myofibroblast-related pathways, including MYOGENESIS, EPITHELIAL_MESENCHYMAL_TRANSITION, and APICAL_JUNCTION (Figure 3B). In contrast, the *SLC39A8*/*SLC39A14*-high group exhibited an enrichment of immune-related and proliferative pathways. These findings further support the association between ZIP transporter expression, particularly *SLC39A7* and *SLC39A13*, and myofibroblastic activation in CAFs.

### 3.4. ZIP Transporter-Based Stratification Defines Prognostic and Immunological Heterogeneity in HNSCC

**Figure 4:**
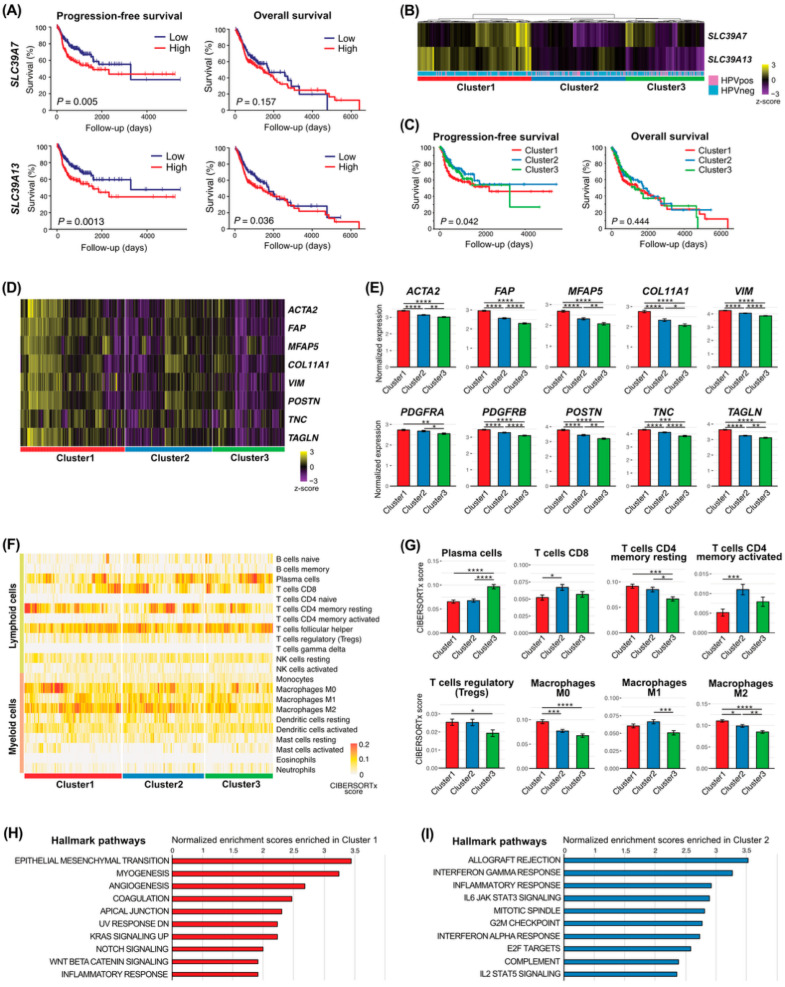
ZIP transporter-based stratification defines prognostic and immunological heterogeneity in HNSCC. (**A**) Kaplan–Meier curves showing progression-free survival and overall survival stratified by *SLC39A7* and *SLC39A13* expression levels. (**B**) Heatmap showing hierarchical clustering of patients based on *SLC39A7* and *SLC39A13* expression, with annotation of HPV status. (**C**) Kaplan–Meier curves showing progression-free survival and overall survival across ZIP-defined clusters. (**D**) Heatmap showing the expression of CAF activation-related genes across ZIP-defined clusters. (**E**) Bar plots showing normalized expression of CAF activation markers across ZIP-defined clusters. (**F**) Heatmap showing immune cell composition estimated by CIBERSORTx across ZIP-defined clusters. (**G**) Bar plots showing selected immune cell populations across ZIP-defined clusters. (**H**,**I**) Bar plots showing gene set enrichment analysis results of Hallmark pathways enriched in ZIP-defined clusters. *, *p* < 0.05; **, *p* < 0.01; ***, *p* < 0.001; ****, *p* < 0.0001.

Given the association between ZIP transporter expression and myofibroblastic activation in CAFs, we next investigated the clinical relevance of ZIP transporters in HNSCC using the TCGA cohort. Survival analysis demonstrated that high *SLC39A13* expression was significantly associated with poorer progression-free survival and overall survival (Figure 4A). In contrast, *SLC39A7* expression showed a weaker or non-significant association with survival outcomes. Similarly, *SLC39A8* and *SLC39A14* expression were not significantly associated with survival outcomes (Supplementary Figure S3A).

Patients were stratified into three clusters based on *SLC39A7* and *SLC39A13* expression patterns. Cluster-based analysis further revealed significant differences in progression-free survival among the ZIP-defined groups, whereas differences in overall survival were less pronounced (Figure 4B,C). Clinicopathological characteristics across ZIP-defined clusters are summarized in Supplementary Table S4. Significant differences were observed in HPV status and primary tumor site distribution among clusters. At the molecular level, clusters with high ZIP expression exhibited an increased expression of CAF activation markers, including *ACTA2*, *FAP*, and *COL11A1* (Figure 4D,E), consistent with a myofibroblastic phenotype.

To characterize the tumor immune microenvironment, CIBERSORTx analysis was performed. ZIP-defined clusters showed distinct immune cell compositions, with *SLC39A13*-high clusters characterized by reduced infiltration of cytotoxic immune cells, including CD8^+^ T cells and activated CD4+ memory T cells, and increased immunosuppressive populations, such as regulatory T cells and M2 macrophages (Figure 4F,G; Supplementary Figure S3B).

Consistently, gene set enrichment analysis demonstrated an enrichment of stromal and extracellular matrix-related pathways in ZIP-high clusters, whereas immune-related pathways were enriched in ZIP-low clusters (Figure 4H,I).

### 3.5. SLC39A13 Is an Independent Prognostic Factor in HNSCC

In univariate Cox regression analysis, multiple clinicopathological variables and ZIP transporter expression were associated with survival outcomes (Table 2). Multivariate analysis demonstrated that *SLC39A13* expression remained an independent predictor of poor progression-free survival, whereas other factors lost statistical significance after adjustment.

## 4. Discussion

In this study, we comprehensively investigated the role of zinc transporter expression in the tumor microenvironment of HNSCC, with a particular focus on cancer-associated fibroblasts. By integrating single-cell transcriptomic analysis, bulk RNA sequencing of primary CAFs, and TCGA-based clinical data, we demonstrated that specific ZIP transporters are preferentially expressed in fibroblasts and are closely associated with myofibroblastic activation, immunosuppressive tumor microenvironments, and adverse clinical outcomes. These findings highlight a previously underappreciated link between zinc transport, stromal activation, and immune regulation in HNSCC. Importantly, our findings extend previous CAF and zinc transporter studies by identifying *SLC39A13* as a fibroblast-specific regulator associated with both stromal activation and immune modulation in the tumor microenvironment.

Our single-cell analysis identified fibroblasts as the major ZIP-expressing population, with *SLC39A7*, *SLC39A8*, *SLC39A13*, and *SLC39A14* preferentially enriched in this compartment. Notably, ZIP7 and ZIP13 are intracellular zinc transporters that regulate zinc flux from secretory compartments and modulate signaling pathways rather than simply reflecting zinc uptake [17,27,28]. This property may be particularly relevant to fibroblasts, whose function depends on extracellular matrix production and TGFβ-driven activation [10,29,30].

Among these transporters, *SLC39A13* showed the strongest association with myofibroblastic activation programs. Previous studies have demonstrated that ZIP13 plays a critical role in connective tissue biology and TGFβ signaling, and its deficiency impairs collagen production and SMAD-dependent responses in fibroblasts [17,28,31]. Consistent with these findings, we observed that *SLC39A13* expression correlated with TGFβ signatures, MMP–TIMP balance, and myCAF-ECM programs, and was enriched in myCAF populations. Similarly, *SLC39A7* has been implicated in intracellular zinc signaling and endoplasmic reticulum homeostasis in mesenchymal cells, supporting a role in maintaining stromal activation states [32,33]. These findings suggest that *SLC39A7* may support stromal fitness and secretory capacity, whereas *SLC39A13* may be closely associated with fibrogenic signaling pathways in HNSCC. This distinction may explain why *SLC39A13*, but not *SLC39A7*, showed a significant association with clinical outcomes, as fibrogenic and TGFβ-driven programs are more directly linked to tumor progression and immune modulation. Given the correlative nature of our analyses, *SLC39A13* should be interpreted as both a marker of myofibroblastic activation and a potential regulator, and further functional studies will be required to establish its causal role.

More broadly, the literature linking ZIP transporters to fibroblast biology remains limited, highlighting the significance of our findings. Most prior studies in nonmalignant mesenchymal systems have focused on skin, bone, and connective tissue development, particularly for ZIP7 and ZIP13, whereas analyses in cancer have largely relied on bulk tumor data [8,17,28,32]. Such approaches may underestimate stromal contributions by averaging signals across heterogeneous cell populations. By integrating single-cell and CAF-focused analyses, we demonstrate that fibroblasts represent a dominant ZIP-expressing compartment in HNSCC, providing a mechanistic basis for the observed association between *SLC39A13* expression and clinical outcomes. In addition, emerging evidence suggests the existence of zinc transporter-defined fibroblast subsets in cancer. For example, in lung cancer, a ZIP1-positive CAF subset has been shown to transfer Zn^2+^ to tumor cells through gap junctions and promote chemoresistance [34]. Our findings extend this concept from a ZIP1-centered chemoresistance model to a ZIP7/ZIP13-centered stromal activation model, suggesting that zinc transporter expression may represent a broader organizing principle of CAF heterogeneity.

Another important aspect is prognosis. ZIP transporters have been linked to aggressive tumor phenotypes in multiple cancer types, although their roles are context-dependent [21,33,35,36]. ZIP7 has been associated with tumor growth and poor prognosis in colorectal cancer, glioma, breast cancer, and lung adenocarcinoma, whereas ZIP13 has been implicated in the migration and invasion of ovarian cancer cells [37,38,39,40,41]. These findings highlight the tumor type-specific functions of ZIP transporters across different malignancies. In our HNSCC cohort, *SLC39A13*, rather than *SLC39A7*, was independently associated with poor progression-free survival in HNSCC, suggesting that ZIP13 may represent a clinically dominant stromal regulator in HNSCC.

The immune-related findings are also biologically plausible. Zinc is a key regulator of both innate and adaptive immunity, and zinc transporters influence signaling in multiple immune cell types [42,43,44]. In addition, pan-cancer analyses have demonstrated associations between SLC39 family members and tumor immune microenvironment features, including immune cell infiltration [45]. In HNSCC, TIMER-based analyses further revealed that multiple SLC39 genes correlate with the abundance of diverse immune cell populations, including CD8^+^ T cells, CD4^+^ T cells, macrophages, and dendritic cells, suggesting a potential role of zinc transporters in shaping immune infiltration patterns [45]. In line with these observations, we found that *SLC39A13*-high tumors were characterized by reduced cytotoxic T-cell infiltration and increased immunosuppressive populations. These findings suggest that ZIP transporter-driven stromal programs may contribute to immune exclusion through CAF-mediated remodeling of the tumor microenvironment, either by promoting active immunosuppressive signaling or by creating physical stromal barriers that limit immune cell infiltration.

This study has several limitations. First, our analyses were primarily based on transcriptomic data and require functional validation at the protein and mechanistic levels. Therefore, our findings should be interpreted as hypothesis-generating, and further experimental studies will be necessary to establish causal mechanisms. Second, the single-cell RNA sequencing dataset used in this study included a limited number of samples, which may restrict the generalizability of the findings. Third, the number of primary CAF samples analyzed for bulk RNA sequencing was limited. Fourth, the causal relationship between ZIP transporter activity and immune modulation remains to be elucidated. Fifth, immune cell composition was estimated using computational deconvolution methods, which may be influenced by tumor purity and stromal content and should be interpreted with caution.

## 5. Conclusions

Our study identified ZIP transporters, particularly *SLC39A13*, as key regulators of CAF activation and tumor microenvironmental remodeling in HNSCC. These findings provide new insights into the role of zinc homeostasis in tumor biology and suggest that targeting ZIP transporter-mediated pathways may represent a novel therapeutic strategy for HNSCC.

## Figures and Tables

**Table 1:** Association between ZIP transporter expression in CAFs and clinicopathological features in HNSCC.

Variables		*SLC39A7*	*SLC39A8*	*SLC39A13*	*SLC39A14*
		Mean (SEM)	Mean (SEM)	Mean (SEM)	Mean (SEM)
HPV status					
	Negative	0.350 (0.008)	0.241 (0.008)	0.265 (0.007)	0.356 (0.009)
	Positive	0.282 (0.017)	0.240 (0.020)	0.180 (0.014)	0.175 (0.014)
	*p*-value	0.0004	0.9604	<0.0001	<0.0001
Primary lesion					
	Larynx	0.378 (0.015)	0.255 (0.016)	0.253 (0.013)	0.401 (0.017)
	Oral cavity	0.339 (0.010)	0.235 (0.009)	0.270 (0.009)	0.338 (0.010)
	Oropharynx	0.282 (0.017)	0.240 (0.020)	0.180 (0.014)	0.175 (0.014)
	*p*-value	0.0002	0.5496	<0.0001	<0.0001
T factor					
	T1-2	0.291 (0.012)	0.198 (0.012)	0.191 (0.010)	0.218 (0.011)
	T3-4	0.365 (0.009)	0.266 (0.009)	0.284 (0.008)	0.385 (0.010)
	*p*-value	<0.0001	<0.0001	<0.0001	<0.0001
N factor					
	N0-1	0.332 (0.010)	0.205 (0.009)	0.218 (0.008)	0.299 (0.010)
	N2-3	0.347 (0.012)	0.294 (0.012)	0.297 (0.011)	0.361 (0.012)
	*p*-value	0.2986	<0.0001	<0.0001	<0.0001

CAF, cancer-associated fibroblast; HNSCC, head and neck squamous cell carcinoma; HPV, human papillomavirus; SEM, standard error of the mean.

**Table 2:** Univariate and multivariate survival analyses of OS and PFS in 520 patients with HNSCC.

Variables		Progression-Free Survival			Overall Survival		
		Univariate	Multivariate		Univariate	Multivariate	
		*p*-Value	HR (95% CI)	*p*-Value	*p*-Value	HR (95% CI)	*p*-Value
HPV status (ref: negative)							
	Positive	0.050			0.137		
Primary lesion (ref: Oropharynx)							
	Hypopharynx	0.057			0.058	2.40 (0.58–9.87)	0.226
	Larynx	0.542			0.127	1.47 (0.60–3.59)	0.393
	Oral cavity	0.149			0.045	2.29 (0.99–5.30)	0.053
T factor (ref: T1–2)							
	T3–4	0.001	1.59 (0.97–2.62)	0.066	0.0002	1.91 (1.17–3.13)	0.010
N factor (ref: negative)							
	Positive	0.063			0.037	1.24 (0.85–1.83)	0.267
M factor (ref: M0)							
	M1	0.284			0.003	6.75 (2.35–19.34)	0.0004
TNM stage (ref: I–II)							
	III–IV	0.011	1.38 (0.73–2.62)	0.324	0.010	0.87 (0.44–1.72)	0.691
*SLC39A7* expression (ref: low)							
	High	0.005	1.37 (0.94–1.98)	0.099	0.158		
*SLC39A8* expression (ref: low)							
	High	0.606			0.305		
*SLC39A13* expression (ref: low)							
	High	0.001	1.64 (1.13–2.38)	0.009	0.036	1.21 (0.87–1.68)	0.266
*SLC39A14* expression (ref: low)							
	High	0.107			0.017	1.19 (0.86–1.65)	0.298

OS, overall survival; PFS, progression-free survival; HNSCC, head and neck squamous cell carcinoma; HPV, human papillomavirus; HR, hazard ratio; CI, confidence interval; ref, reference.

## Data Availability

The original data presented in this study are openly available in GSE164690, https://www.ncbi.nlm.nih.gov/geo/query/acc.cgi?acc=GSE164690 (accessed on 1 June 2023) and on the FireBrowse website, http://firebrowse.org/ (accessed on 6 May 2020).

## Supplementary Materials

The following supporting information can be downloaded at: https://www.mdpi.com/article/10.3390/curroncol33050292/s1, Figure S1. Expression patterns of zinc transporter genes across cell types in HNSCC single-cell data. Figure S2. Additional data to Figure 2. Figure S3. Survival analysis and immune cell composition associated with ZIP transporter expression (additional data to Figure 4). Table S1. Clinical characteristics of patients included in the GSE164690 single-cell RNA sequencing dataset. Table S2. Top 20 differentially expressed genes for CAF subtypes. Table S3. Clinicopathological features of 10 patients for bulk RNA sequencing. Table S4. Association between *SLC39A7*/*SLC39A13*-based clusters and clinicopathological features in 520 patients with HNSCC.

## Abbreviations

The following abbreviations are used in this manuscript:

CAF	Cancer-associated fibroblast
DEG	Differentially expressed gene
FDR	False discovery rate
GEO	Gene Expression Omnibus
GSEA	Gene set enrichment analysis
HNSCC	Head and neck squamous cell carcinoma
HPV	Human papillomavirus
iCAF	Inflammatory cancer-associated fibroblast
myCAF	Myofibroblastic cancer-associated fibroblast
NES	Normalized enrichment score
OS	Overall survival
PFS	Progression-free survival
ROC	Receiver operating characteristic
scRNA-Seq	Single-cell RNA sequencing
TME	Tumor microenvironment
UMAP	Uniform Manifold Approximation and Projection
