# Supplementary material for: SLC39A13 Defines Myofibroblastic Activation and Immunosuppressive Tumor Microenvironment in Head and Neck Squamous Cell Carcinoma
Source: Curr Oncol. 2026 May 18;33(5):292. doi: 10.3390/curroncol33050292 (PMC13205769; doi:10.3390/curroncol33050292)
Supplement: Supplementary file 1 [file curroncol-33-00292-s001.zip › curroncol-4272369-supplementary.pdf]

Supplementary Figures

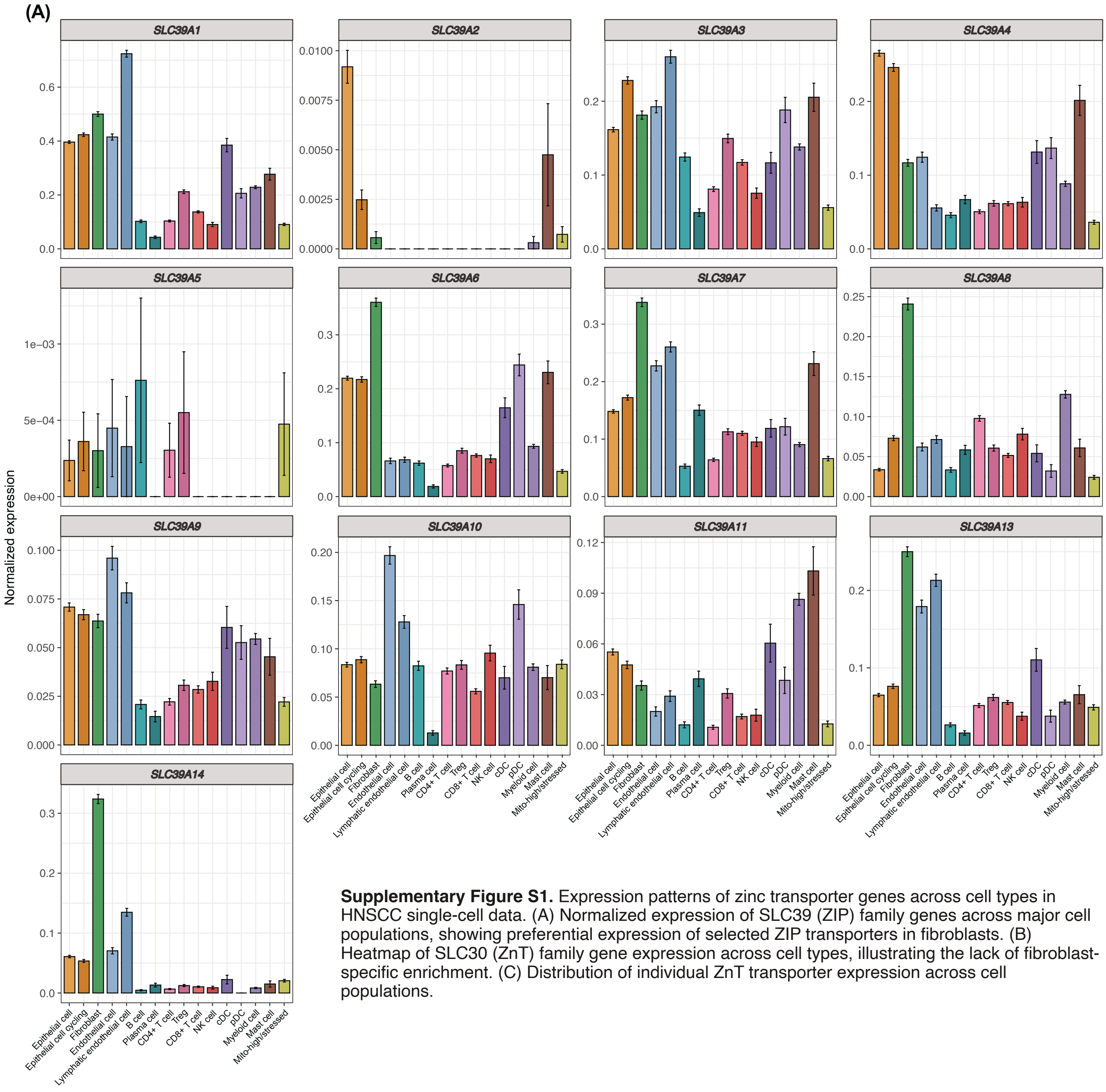

Supplementary Figure S1. Expression patterns of zinc transporter genes across cell types in HNSCC single-cell data. (A) Normalized expression of SLC39 (ZIP) family genes across major cell populations, showing preferential expression of selected ZIP transporters in fibroblasts. (B) Heatmap of SLC30 (ZnT) family gene expression across cell types, illustrating the lack of fibroblast-specific enrichment. (C) Distribution of individual ZnT transporter expression across cell populations.

Supplementary Figures

(B)

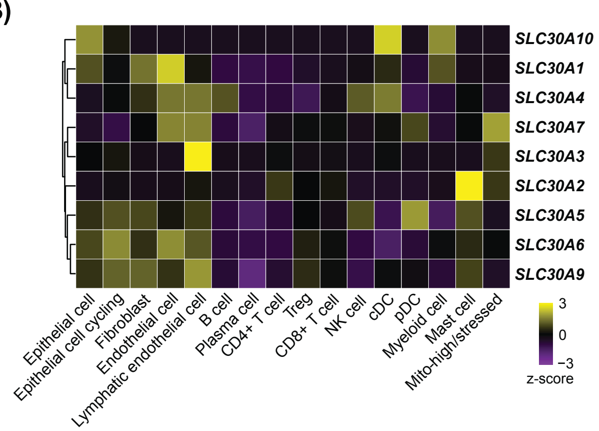

(C)

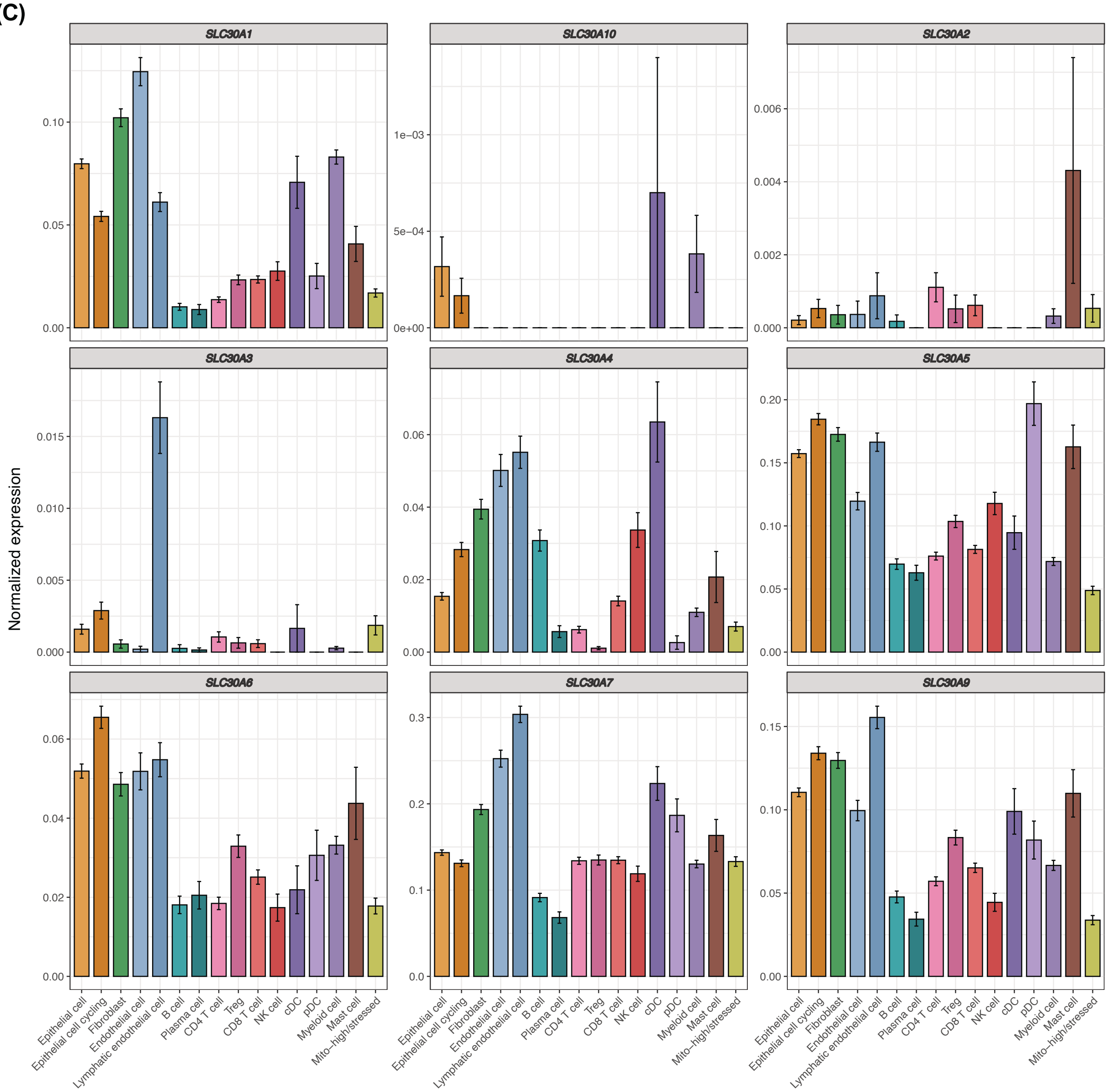

Supplementary Figures

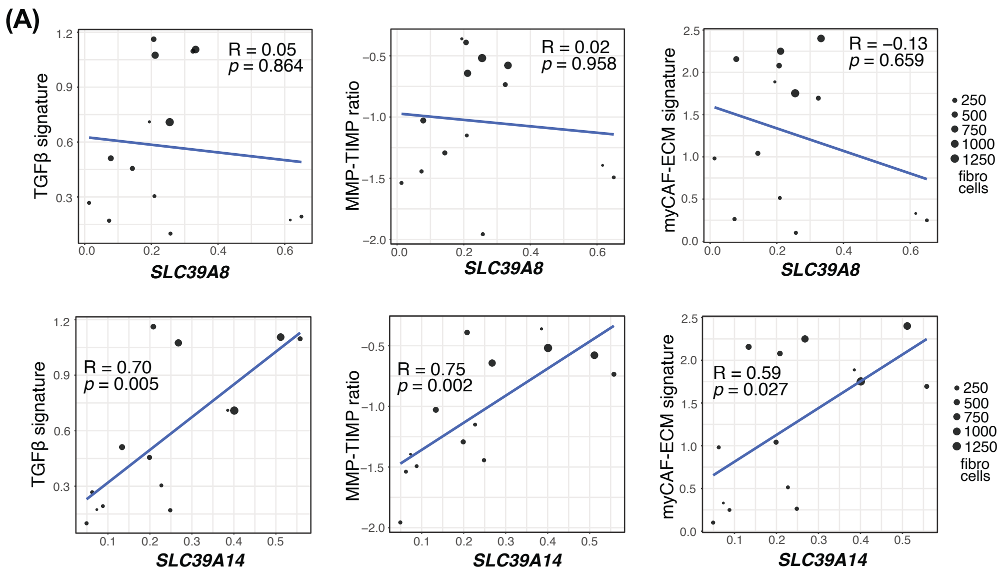

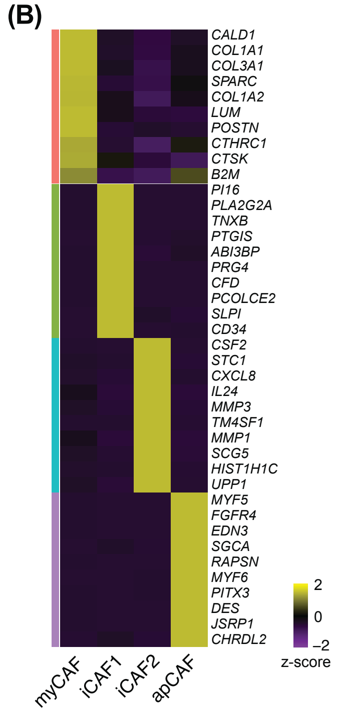

Supplementary Figure S2. Additional data to Figure 2.
(A) Correlation between ZIP transporter expression and stromal activation signatures in fibroblasts. (B) Heatmap showing the expression of top 10 differentially expressed genes in CAF subtypes (myCAF, iCAF1, iCAF2, and apCAF).

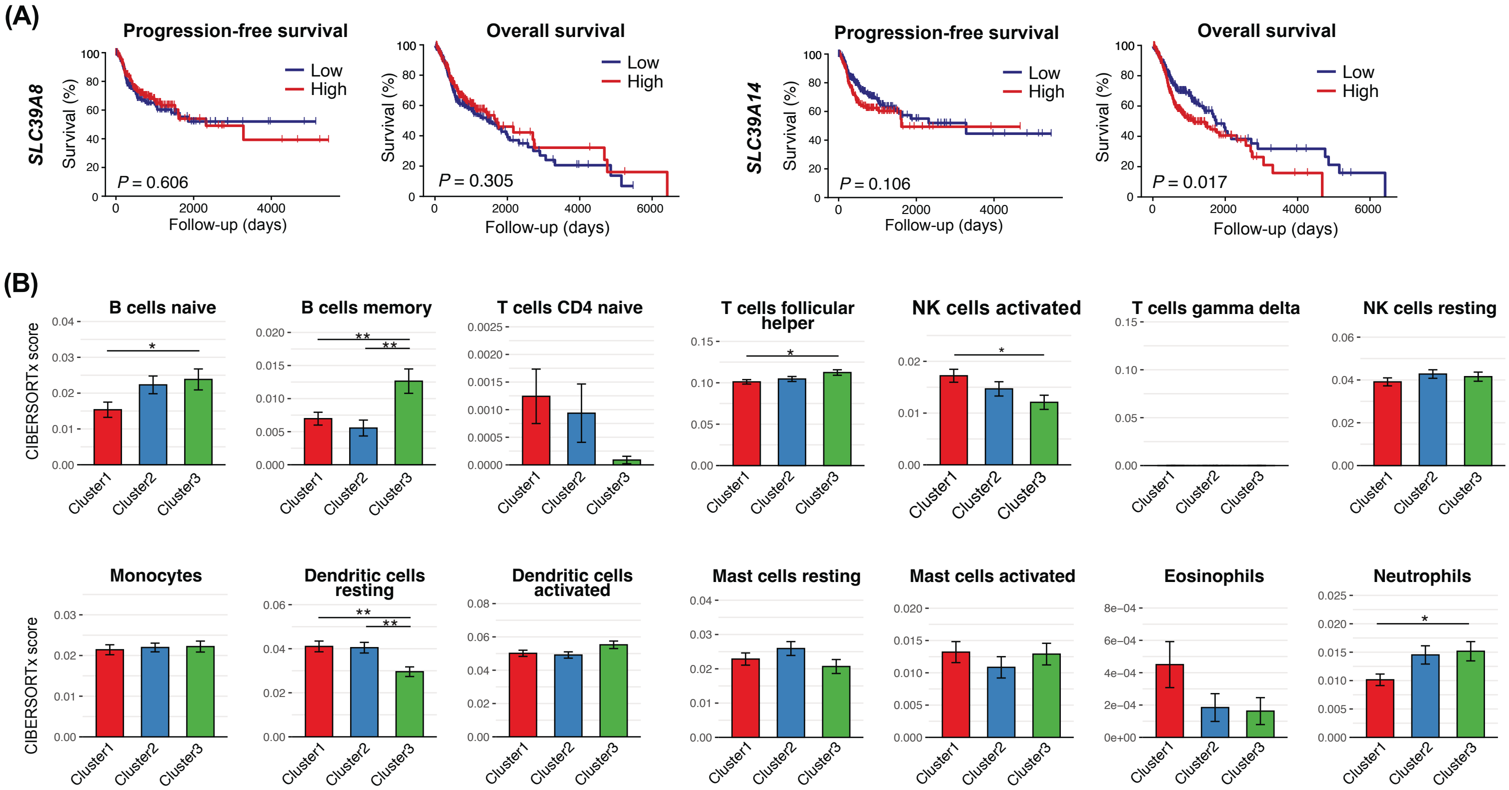

Supplementary Figure S3. Survival analysis and immune cell composition associated with ZIP transporter expression (additional data to Figure 4). (A) Kaplan–Meier curves for overall survival and progression-free survival stratified by *SLC39A8* and *SLC39A14* expression levels. (B) Comparison of immune cell fractions estimated by CIBERSORTx across ZIP-defined clusters, including B cells, T cells, NK cells, dendritic cells, mast cells, eosinophils, and neutrophils.

Supplementary Table

Supplementary Table S1. Clinical characteristics of patients included in the GSE164690 single-cell RNA sequencing dataset.

Patient	Sex	Age group	Smoking	Alcohol	Disease site	T factor	N factor	M factor	HPV status
1	M	70-79	Yes	No	Oral cavity	T4A	N2B	M0	Neg
5	F	50-59	Yes	Yes	Oral cavity	T3	N3b	M0	Neg
6	M	30-39	Yes	Yes	Oral cavity	T3	N0	M0	Neg
7	M	60-69	Yes	Yes	Larynx	T3	N0	M0	Neg
8	F	70-79	Yes	Yes	Oral cavity	T1	N0	M0	Neg
9	F	70-79	Yes	Yes	Oral cavity	T3	N2B	M0	Neg
11	M	80-89	No	No	Oral cavity	T2	N0	M0	Neg
12	M	50-59	Yes	Yes	Oropharynx	T2	N1	M0	Pos
13	M	70-79	No	No	Oropharynx	T2	N0	M0	Pos
14	M	50-59	Yes	Yes	Oropharynx	T1	N1	M0	Pos
15	F	60-69	Yes	NA	Oral cavity	T2	N0	M0	Neg
16	M	40-49	Yes	Yes	Oropharynx	T2	N1	M0	Pos
17	M	50-59	Yes	Yes	Oropharynx	T1	N1	M0	Pos
18	M	50-59	Yes	Yes	Oropharynx	T2	N2	M0	Pos

Supplementary Table S2. Top 20 differentially expressed genes for CAF subtypes.

**myCAF**	**iCAF1**	**iCAF2**	**apCAF**
SPARC	*RARRES2*	*MMP1*	*APOC1*
SPON2	*IDO1*	*CXCL8*	*APOE*
BGN	*VCAM1*	*C15orf48*	*HLA-DRA*
POSTN	*CCL2*	*FTH1*	*CLU*
SERPINH1	*CD74*	*FTL*	*HLA-DRB1*
CTHRC1	*CXCL10*	*IL24*	*RBP1*
SNAI2	*TWIST2*	*ADM*	*ID3*
MYL9	*TMEM176B*	*SCG5*	*MEST*
PCOLCE	*DPT*	*IGFBP3*	*CD74*
LOXL1	*KLF4*	*CREG1*	*BTG2*
CLEC11A	*RGCC*	*ANGPTL4*	*MSC*
THY1	*CXCL12*	*MT2A*	*DNAJB1*
PRSS23	*GBP1*	*HAS2*	*MT1X*
TPM1	*DUSP1*	*STC1*	*PPP1R14B*
TAGLN	*TSC22D1*	*STC2*	*CHI3L1*
IL7R	*CEBPD*	*RND3*	*ATP1B1*
ACTA2	*CXCL13*	*IL6*	*SELENOP*
MEST	*ISG15*	*CXCL3*	*CXCL2*
MMP9	*CLU*	*PLIN2*	*TUBB2B*
MMP11	*CXCL2*	*HIST1H1C*	*FABP5*

Supplementary Table

Supplementary Table S3. Clinicopathological features of 10 patients for bulk RNA sequencing.

Case	Sex	Age	Disease site	T factor	N factor	M factor	Stage	HPV status
CAF1	M	72	Hypopharynx	3	2b	0	IVA	Negative
CAF2	M	44	Oropharynx	2	1	0	I	Positive
CAF3	M	69	Hypopharynx	4a	3b	0	IVB	Negative
CAF4	M	77	Oropharynx	2	1	0	I	Positive
CAF5	M	60	Oropharynx	2	0	0	I	Positive
CAF6	M	80	Oropharynx	2	1	0	I	Positive
CAF7	M	50	Oropharynx	2	1	0	I	Positive
CAF8	M	74	Hypopharynx	3	2b	0	IVA	Negative
CAF9	M	67	Hypopharynx	4a	2b	0	IVB	Negative
CAF10	M	74	Hypopharynx	4a	2b	0	IVB	Negative

Supplementary Table S4. Association between *SLC39A7 /SLC39A13*-based clusters and clinicopathological features in 520 patients with HNSCC.

Variables	Cluster 1 n (%)	Cluster 2 n (%)	Cluster 3 n (%)	*P*-value
HPV status				
Negative	190 (92.2)	130 (76.0)	103 (72.0)	<0.0001
Positive	16 (7.8)	41 (24.0)	40 (28.0)	
Primary lesion				
Hypopharynx	6 (2.9)	2 (1.2)	2 (1.4)	<0.0001
Larynx	32 (15.5)	36 (21.1)	48 (33.6)	
Oral cavity	160 (77.7)	116 (67.8)	75 (52.4)	
Oropharynx	8 (3.9)	17 (9.9)	18 (12.6)	
T factor				
T1-2	86 (41.7)	68 (39.8)	56 (39.2)	0.872
T3-4	120 (58.3)	103 (60.2)	87 (60.8)	
N factor				
Negative	77 (39.5)	67 (41.6)	69 (51.1)	0.096
Positive	118 (60.5)	94 (58.4)	66 (48.9)	
M factor				
M0	202 (98.1)	168 (98.8)	134 (97.8)	0.127
M1	0 (0.0)	2 (1.2)	3 (2.2)	
TNM stage				
I-II	46 (22.3)	36 (21.1)	29 (20.3)	0.894
III-IV	160 (77.7)	135 (78.9)	114 (79.7)	
